# Neutralizing SARS-CoV-2 Antibodies in Commercial Immunoglobulin Products Give Patients with X-Linked Agammaglobulinemia Limited Passive Immunity to the Omicron Variant

**DOI:** 10.1007/s10875-022-01283-9

**Published:** 2022-05-11

**Authors:** Hannes Lindahl, Jonas Klingström, Rui Da Silva Rodrigues, Wanda Christ, Puran Chen, Hans-Gustaf Ljunggren, Marcus Buggert, Soo Aleman, C. I. Edvard Smith, Peter Bergman

**Affiliations:** 1 Department of Clinical Immunology and Transfusion Medicine, Karolinska University Hospital, Stockholm, Sweden; 2 Department of Clinical Neuroscience, Karolinska Institutet, Stockholm, Sweden; 3 Department of Medicine Huddinge, Karolinska Institutet, Stockholm, Sweden; 4 Department of Infectious Diseases, Karolinska University Hospital, Stockholm, Sweden; 5 Department of Laboratory Medicine, Karolinska Institutet, Stockholm, Sweden

**Keywords:** Primary immunodeficiency, Immunoglobulin replacement therapy, SARS-CoV-2, Omicron, Passive immunity, X-linked agammaglobulinemia

## Abstract

Immunodeficient individuals often rely on donor-derived immunoglobulin (Ig) replacement therapy (IGRT) to prevent infections. The passive immunity obtained by IGRT is limited and reflects the state of immunity in the plasma donor population at the time of donation. The objective of the current study was to describe how the potential of passive immunity to SARS-CoV-2 in commercial off-the-shelf Ig products used for IGRT has evolved during the pandemic. Samples were collected from all consecutive Ig batches (*n* = 60) from three Ig producers used at the Immunodeficiency Unit at Karolinska University Hospital from the start of the SARS-CoV-2 pandemic until January 2022. SARS-CoV-2 antibody concentrations and neutralizing capacity were assessed in all samples. In vivo relevance was assessed by sampling patients with XLA (*n* = 4), lacking endogenous immunoglobulin synthesis and on continuous Ig substitution, for plasma SARS-CoV-2 antibody concentration. SARS-CoV-2 antibody concentrations in commercial Ig products increased over time but remained inconsistently present. Moreover, Ig batches with high neutralizing capacity towards the Wuhan-strain of SARS-CoV-2 had 32-fold lower activity against the Omicron variant. Despite increasing SARS-CoV-2 antibody concentrations in commercial Ig products, four XLA patients on IGRT had relatively low plasma concentrations of SARS-CoV-2 antibodies with no potential to neutralize the Omicron variant in vitro. In line with this observation, three out the four XLA patients had symptomatic COVID-19 during the Omicron wave. In conclusion, 2 years into the pandemic the amounts of antibodies to SARS-CoV-2 vary considerably among commercial Ig batches obtained from three commercial producers. Importantly, in batches with high concentrations of antibodies directed against the original virus strain, protective passive immunity to the Omicron variant appears to be insufficient.

## Introduction

Individuals with primary immunoglobulin (Ig) deficiencies have reduced or absent antibody responses after infection or vaccination. For many of these patients, long-term Ig replacement therapy (IGRT) is a life-saving treatment that also prevents lung damage resulting from recurrent respiratory tract infections [1]. The Ig preparations used for IGRT are made from plasma pools collected from at least 1000 healthy donors, and each batch consequently reflects the immune status of this population at the time of donation. The time from donation to production is typically in the range of 6–9 months, which explains why the antibody content in IGRT products used clinically does not fully reflect the current serostatus of the population for an emerging pathogen.

Since the start of the severe acute respiratory syndrome coronavirus 2 (SARS-CoV-2) pandemic, the role of donor-derived Ig has been discussed, either as immunomodulation or a source of cross-reactive virus-neutralizing antibodies [2, 3]. However, it has been reported that the cross-reactive antibodies in pre-pandemic Ig batches are non-neutralizing [4–6]. Because of the increasing SARS-CoV-2 seroprevalence within the plasma donor population, the emergence of SARS-CoV-2 specific neutralizing antibodies in Ig products has been anticipated. Recently, plasma industry representatives have reported the appearance of neutralizing SARS-CoV-2 antibodies in commercial Ig batches starting in September 2020 and with a rapid increase in the concentration thereafter, especially after vaccination against SARS-CoV-2 became widely available [7–9]. We here report the largest academia-initiated assessment of neutralizing SARS-CoV-2 antibodies encompassing all consecutive IGRT batches (*n* = 60) used at the Immunodeficiency Unit in the Karolinska University Hospital since the start of the pandemic.

Patients registered at the Immunodeficiency Unit outpatient clinic for IGRT receive the prophylactic treatment either as subcutaneous (SCIG) or intravenous (IVIG) infusions. We hypothesized that SARS-CoV-2 antibodies in SCIG or IVIG products would increase over time as a consequence of natural disease or vaccination among the donors. To test this hypothesis, we collected aliquots of all batches of Ig preparations used at our outpatient clinic and assessed the content for wild-type/Wuhan-strain spike (S1) IgG and neutralizing activity against viable SARS-CoV-2. The presence of neutralizing antibodies in commercial preparations could tentatively give some degree of passive immunity in patients with antibody deficiencies with implications for patient management. Most of the patients with antibody deficiencies at our center have now been vaccinated against SARS-CoV-2, with 2 or 3 doses. The serological response was poor for patients with common variable immunodeficiency (CVID) and completely absent for those with X-linked agammaglobulinemia (XLA) [10]. Thus, even a partly protective effect of IGRT can have direct clinical benefits for these patients.

## Materials and Methods

### SARS-CoV-2 Serology

SARS-CoV-2 anti-wild-type spike 1 (S1) antibody concentration was primarily assessed using the Phadia System (Thermo Fisher Scientific, Waltham, MA, USA). For diagnostic use on serum samples, a value above 10 U/ml is considered a positive result and 7–10 U/ml as borderline, according to the manufacturer. A subset of samples was also analyzed using the Bio-Plex SARS-CoV-2 serology assay kit (Bio-Rad, Hercules, CA, USA). Production of IVIG or SCIG involves a several-fold enrichment of IgG content compared to that in donor plasma and the concentrations for the products included in this study ranged from 100 to 200 mg/ml. To make results comparable across different products, data were adjusted so that they corresponded to a total IgG concentration of 100 mg/ml; e.g., SARS-CoV-2 antibody concentrations from products with 200 mg/ml of total IgG were divided by 2. IVIG/SCIG producers represented were CSL Behring (King of Prussia, PA, USA), Octapharma (Lachen, Switzerland), and Takeda (Tokyo, Japan).

### SARS-CoV-2 Neutralization Assay

Live-virus microneutralization assay based on cytopathic effects (CPE) with SARS-CoV-2 wild type and Omicron strains was performed as previously described [11, 12], and samples were twofold serially diluted. Similar to the serology results, a conversion factor was applied to neutralization titers to make results from products with different total IgG concentration (100–200 mg/ml) comparable.

### Plasma Samples

The present study used samples and data from four individuals with XLA and four healthy controls that were collected for a clinical trial previously described [10]. Use of these samples and data was approved by the Swedish Ethical Review Authority (ID2021-00,451). All participants provided written informed consent.

## Results

### SARS-CoV-2 Serology

**Fig. 1:**
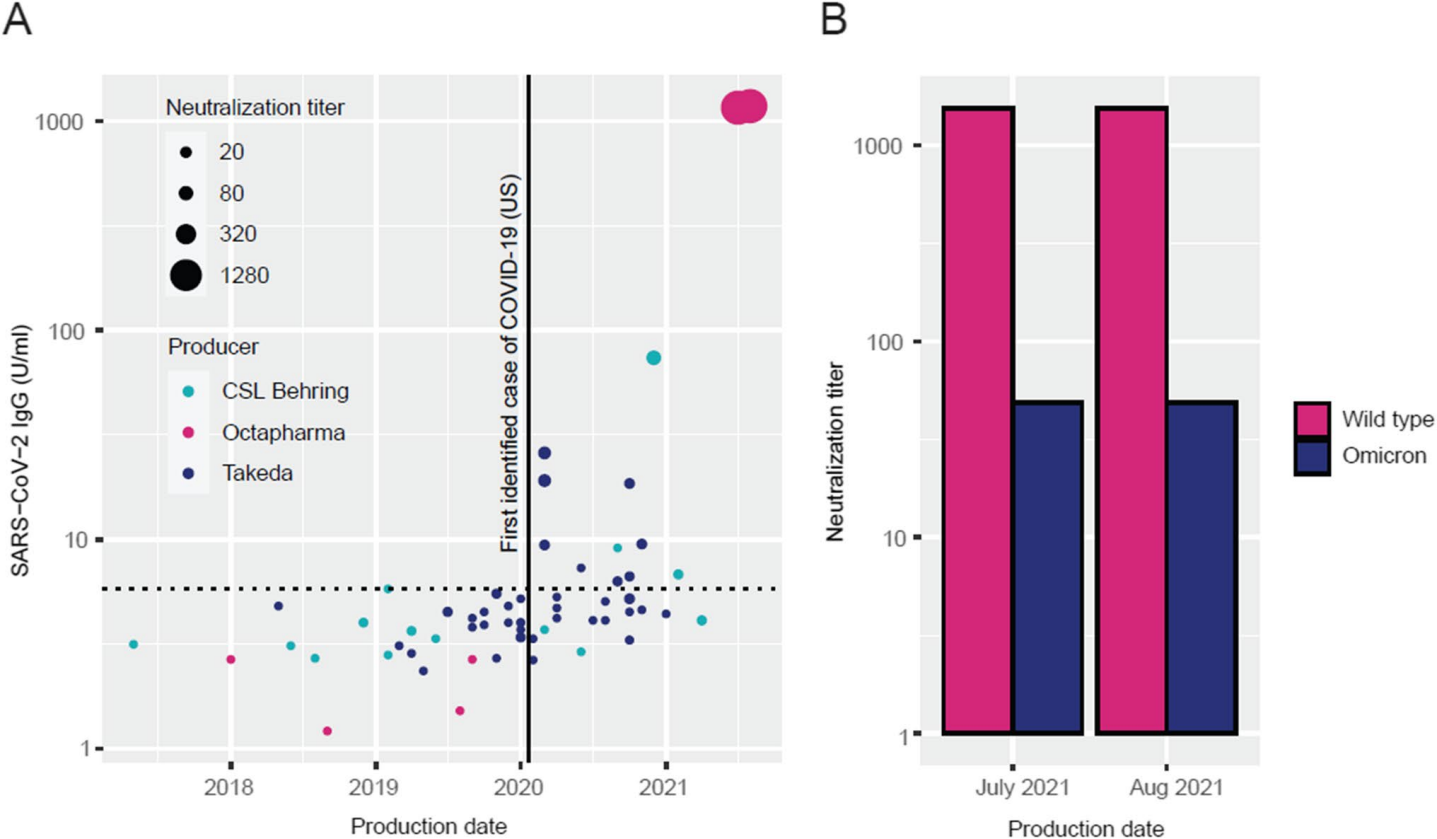
The emergence of neutralizing antibodies against wild-type and Omicron variants of SARS-CoV-2 in commercial immunoglobulin products. **A** Anti-SARS-CoV-2 spike protein antibody concentration and neutralizing capacity in consecutive batches of commercial immunoglobulin products used in clinical practice at the Immunodeficiency Unit at Karolinska University Hospital in relation to production date. The size of the dots represents the highest dilution (titer) at which the individual batch is effective in a microneutralization assay using wild-type/Wuhan strain live SARS-CoV-2. The producers are represented by different colors. In pre-pandemic batches (left of vertical line) no antibody concentration above 5.8 U/ml was observed, which is represented by a dashed horizontal line. Concentrations and titers have been adjusted to compensate for differences in total IgG concentration (100–200 g/l) in the included products. **B** The two immunoglobulin batches with the highest concentration of antibodies and neutralizing capacity against wild-type SARS-CoV-2 were also tested for cross-reactive neutralization capacity against the Omicron variant of SARS-CoV-2

A total of 60 Ig batches were collected with production dates ranging from May 2017 to August 2021 and were analyzed for anti-SARS-CoV-2 S1 antibody concentration using the Phadia System. Of the 30 batches produced in January 2020 or earlier, i.e., before the first identified COVID-19 case in the USA, no value above 5.8 U/ml was detected (Fig. 1A). Of the 30 batches produced after this date, 13 had values above 5.8 U/ml (Fisher’s exact test, *p* =  < 0.00001). Notably, although the frequency of seropositive batches increased over time, several batches produced during 2021 did not show the presence of specific antibodies to SARS-CoV-2.

We reanalyzed a subset of samples (*n* = 23) for wild-type anti-S1 antibody concentration using the Bio-Plex SARS-CoV-2 serology assay and observed a good linear correlation in positive samples and coherence regarding classification as negative/positive when the threshold for positive in the Bio-Plex assay was set at 6 U/ml. As a quality control of general antibody integrity in these Ig batches, which in many cases were stored beyond the date of expiration, we also quantified anti-pneumococcal capsular polysaccharide IgG2 in a subset (*n* = 37). No deterioration over time could be observed suggesting that the quality was maintained (data not shown).

### Live SARS-CoV-2 Neutralization

The Phadia SARS-CoV-2 serology assay, and other commonly used serological assays for SARS-CoV-2, detects antibodies that bind to the S1 subunit of the spike protein located on the surface of the virus. The amounts of antibodies identified in this type of assay reflect exposure to the virus and vaccination but do not necessarily reflect the capacity of these antibodies to prevent infection. We tested all collected Ig batches in a live microneutralization assay against infectious wild-type SARS-CoV-2 and, as expected, found the greatest neutralizing capacity in batches produced from plasma collected after the start of the pandemic and observed higher neutralization activity in batches with higher antibody concentrations (Fig. 1A). Of note, like the SARS-CoV-2 antibody concentrations detected by serological assays, a proportion of Ig batches produced late in the pandemic had no detectable neutralizing activity.

The first reports of the SARS-CoV-2 Omicron variant came in November 2021 and it is currently the dominating variant [13]. The heavily mutated Omicron variant has been designated as a variant of concern (VOC) by the World Health Organization and reports confirm the predicted loss of effective immunity from previous infection or vaccination [14–16]. From the collected Ig batches, the two with the highest capacity to neutralize the Wuhan strain of the virus were tested against the Omicron variant in the same neutralization assay and we observed a 32-fold lower potency against Omicron in both batches (Fig. 1B). The plasma for these Ig batches was collected long before the Omicron variant emerged, and neutralization therefore reflects antibody cross-reactivity.

**Fig. 2:**
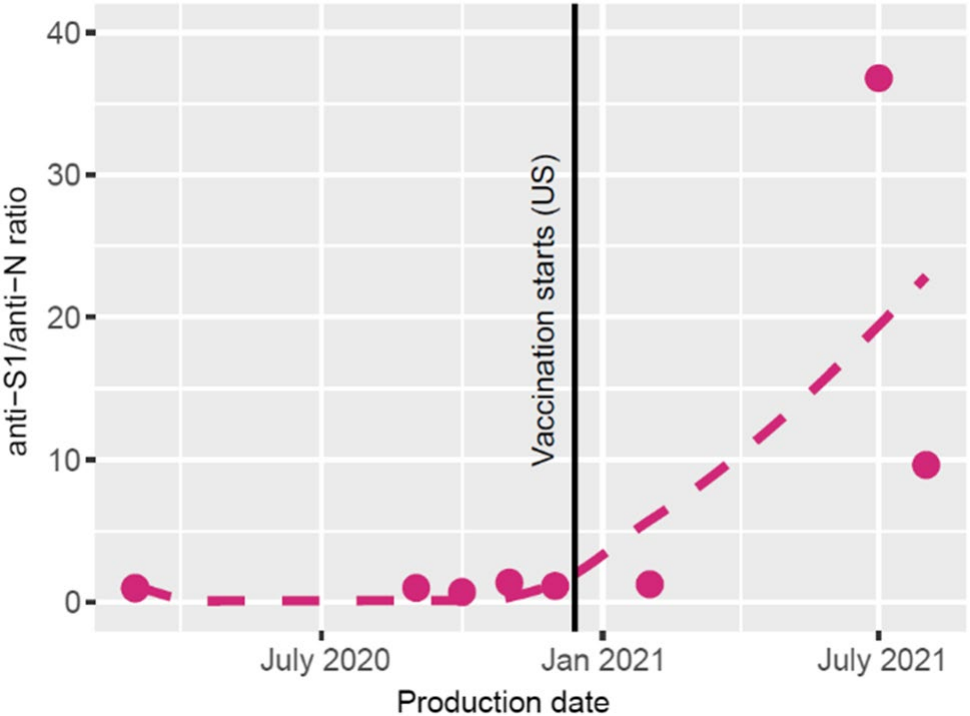
Increasing anti-spike 1/anti-nucleocapsid antibody ratio over time in commercial immunoglobulin products correlates with SARS-CoV-2 vaccination of the plasma donor population. The date SARS-CoV-2 vaccination started in the USA is indicated (vertical black line). A fitted line (dotted) represents a rough estimate of the change in anti-spike 1 (S1)/anti-nucleocapsid (N) antibody ratio after this date

Detection of antibodies to SARS-CoV-2 nucleocapsid (N) reflects immunity after infection while anti-S1 reflects immunity after either infection or vaccination. By analyzing positive samples for both antibody specificities, we could confirm a proportional increase in anti-S1 antibodies in Ig batches produced after vaccination was initiated in the US population (Fig. 2).

### Donor-Derived Antibodies in XLA Patient Plasma

**Fig. 3:**
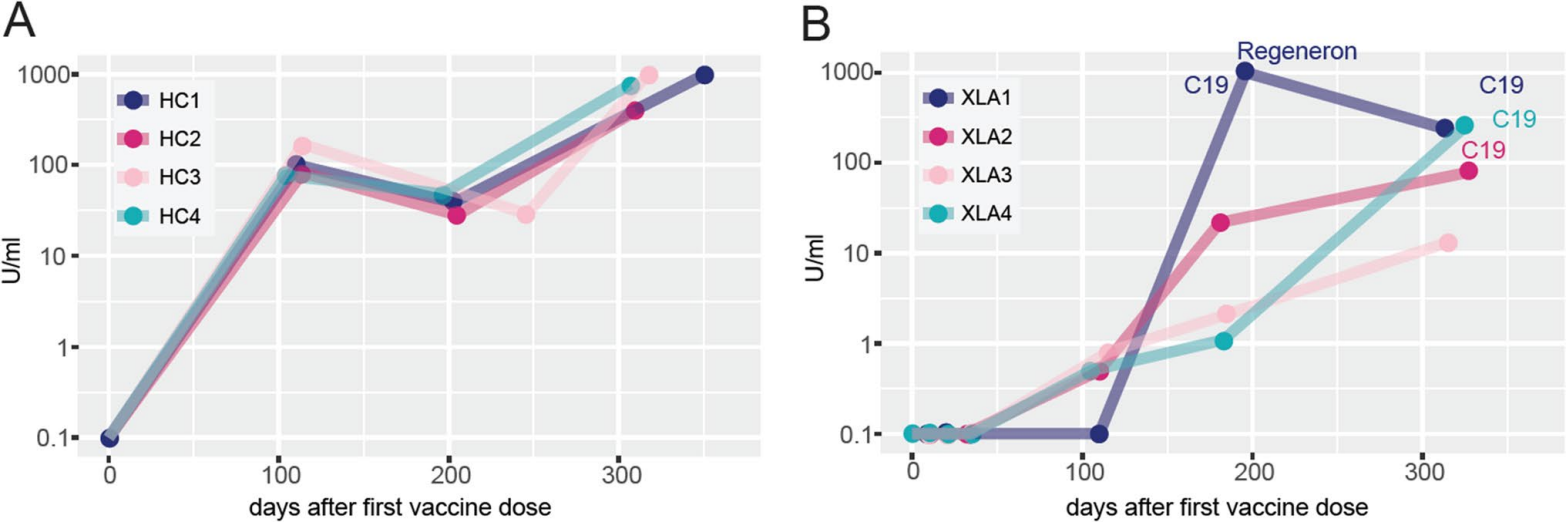
SARS-CoV-2 antibody concentrations after first vaccine dose in XLA patients on continuous immunoglobulin replacement therapy. **A** Four healthy controls and **B** four XLA patients were given the first and second doses of mRNA BNT162b2 vaccine against SARS-CoV-2 on days 0 and 21, respectively. All had an additional dose before their final plotted value. Anti-spike antibody levels were determined using the Phadia system except for XLA2 day 327 and XLA4 day 344 for which the Thermo system was used. PCR-positive COVID-19 (C19) in the XLA patients is indicated using the corresponding color. XLA1 had been treated with SARS-CoV-2-specific monoclonal antibodies (Regeneron) before the antibody assessment on day 196. *HC* healthy control, *XLA* X-linked agammaglobulinemia

Four XLA patients with no endogenous antibody production were monitored prospectively as part of an ongoing clinical trial (COVAXID) studying the response to the Pfizer-BioNTech BNT162b2 mRNA vaccine against SARS-CoV-2 in patients with primary immunodeficiencies (Table 1) [10]. They received their first vaccine dose between February 18 and March 8, 2021, and owing to their genetic defect, did not seroconvert within the first 35 days (Fig. 3). However, 3 to 6 months later all four had detectable but low antibody responses. These patients are on continuous IGRT and we interpret the observed seroconversion as an effect of recently emerged SARS-CoV-2 antibodies in the Ig batches that they had received. Notably, one of these patients (XLA1) had COVID-19 before the planned sampling at 6 months and was treated with the SARS-CoV-2 monoclonal antibody drug Regeneron and consequently had much higher serological results compared to the others. The highest plasma concentration of SARS-CoV-2 antibodies detected in the other three XLA patients were 250 U/ml, which in our experience can be titrated to approximately 1:250 before losing neutralizing activity to the Wuhan strain in vitro. However, antibody concentrations at this level are too low to have any potential to neutralize the Omicron variant in vitro, if we assume that there is the same (32-fold) loss in efficiency compared to the Wuhan strain that we observed for commercial donor-derived plasma products (Fig. 1B). Moreover, three of them recently had symptomatic COVID-19 during the Omicron wave.

**Table 1:** XLA patient clinical characteristics and SARS-CoV-2-related data

	XLA1	XLA2	XLA3	XLA4
Age	39	47	48	38
Sex	Man	Man	Man	Man
Age of onset	6 months	Early childhood	Early childhood	Early childhood
Comorbidities	None	TBE sequele (cognitive)	CD, T cell lymphoma	Bronchiectasis
IGRT product (producer)	Hyqvia (Takeda)	Hizentra (CSL Behring)	Hizentra	Hizentra
IGRT dose (g/week)	45	16	20	10
Immunomodulatory treatment	None	None	Budesonid	None
Prophylactic anti-infectives	None	None	None	Azithromycin
Frequency RTI (per year)	3–4	3–4	3–4	1–2
FEV_1_ (% of expected)	77	113	NA	94
*BTK* variant				
Location	Intron 18 to exon 19 [22]	Intron 4 [23, 24]	Exon 14 [22]	Exon19 [25]
Type of variant	3042-bp deletion	Splice site	Nonsense	Missense
Nucleotide change/consequence	All of exon 19 lost	Includes pseudo-exon 4a, frameshift	p.Trp421*	p.Arg641His
Domain	Kinase	PH	Kinase	Kinase
Latest lab results				
IgG (g/l)	14.2	12.8	11.3	14.8
IgM (g/l)	< 0.06	< 0.06	2.29 g/l (M-component)	< 0.06
IgA (g/l)	< 0.07	< 0.07	< 0.03	< 0.07
B cells (10^9^/l)	< 0.01	< 0.01	< 0.01	NA
T cells (10^9^/l)	0.67	1.14	1.0	NA
NK cells (10^9^/l)	0.21	0.11	0.02	NA
COVID-19				
COVID-19 after vaccination	Yes/Yes	Yes	No	Yes
Days after vaccine dose 1	166/340	327	-	344
Severity	Mild/Mild	Mild	-	Mild
Symptoms	Upper RTI, fever (both times)	Upper RTI, no fever	-	Upper RTI, fever
SARS-CoV-2 genotype	B.1.617.2 (Delta)/NA	BA.1 (Omicron)	-	NA
COVID-19 treatment (days after vaccine dose 1)	Regeneron (167)	No	No	No

Analyses [reference range]: IgG [6.7–14.5 g/l], IgM [0.27–2.10 g/l], IgA [0.88–4.50], B cells [0.08–0.28], T cells [0.65–1.57 (10^9^/l)], NK cells [0.1–0.35 (10^9^/l)]

*XLA* X-linked agammaglobulinemia, *TBE* tick-borne encephalitis, *CD* Crohn’s disease, *NA* not available, *BTK* Bruton tyrosine kinase, *bp* base pairs, *PI* post-vaccination, *RTI* respiratory tract infection, *FEV*_*1*_ forced expiratory volume in 1 s

## Discussion

We have assessed SARS-CoV-2 antibody concentrations and neutralizing activity in all consecutive batches of commercial Ig used for our patients at the Immunodeficiency Unit in Karolinska University Hospital up until January 2022. The objective was to assess the presence of antibodies with potential to reduce the impact of SARS-CoV-2 on patients with primary IgG deficiencies.

While the importance of antibodies for protective immunity to pathogens in general is undisputed, the immunological factors that mitigate the consequences of COVID-19 in patients with antibody deficiencies are not well understood [17]. Although the disorder XLA precludes any antibody response after vaccination, there is a solid rationale to vaccinate these patients since the resulting T cell immunity is assumed to be beneficial after infection. In fact, T cell immunity against SARS CoV-2 in patients with XLA have been shown to be similar or even better than in healthy controls [18]. It should be noted, however, that a T cell response, without neutralizing antibodies, may be insufficient to fully clear the virus from the host [19]. Administration of donor-derived SARS-CoV-2 antibodies to patients with primary or secondary immunodeficiencies has appeared efficacious in selected cases [20, 21], but the effects of SARS-CoV-2 antibodies in IGRT products for antibody-deficient patients with or without specific T cell immunity are not known.

We conclude that 2 years into the SARS-CoV-2 pandemic neutralizing antibodies are not consistently detected in commercially available Ig products. Moreover, Ig batches with high anti-SARS-CoV-2 antibody concentrations because of past infection and vaccination among plasma donors do not efficiently neutralize the Omicron variant resulting in symptomatic infection in seropositive XLA patients for whom IGRT has been the sole source of any circulating antibodies. These results may influence the risk assessments for patients on IGRT and can also serve as a model for future pandemics to forecast the delay until passive immunity from standard Ig preparations can be expected. Interestingly, the levels of anti-S1 IgG in XLA patients on continuous IGRT appear to be at approximately the same level as healthy controls after 2 vaccine doses. This is clearly not enough to neutralize the Omicron variant, neither in healthy individuals nor in these patients. However, it is likely that continued vaccination in society with additional doses will raise the SARS-CoV-2 antibody content and neutralizing activity of donor plasma. This could subsequently improve the protective capacity of IGRT products against the Omicron variant, with potential benefit to antibody-deficient patients.

## Data Availability

Data availability is specified in the original study [10].
